# The impact of preoperative stroke on 1-year mortality and days at home alive after major surgery: an observational cohort study

**DOI:** 10.1186/s13741-024-00453-0

**Published:** 2024-10-04

**Authors:** Matilda Widaeus, Alva Cedermark, Max Bell

**Affiliations:** 1 Department of Anaesthesia and Intensive Care Medicine, Karolinska University Hospital, Stockholm, Sweden; 2 Department of Physiology and Pharmacology, Karolinska Institute, Stockholm, Sweden

**Keywords:** Preoperative stroke, Cerebrovascular disease, Surgery, Risk factors

## Abstract

**Objective:**

The lifetime risk of stroke is one in four people. As the population aged over 60 constantly expands, the impact of stroke on perioperative care is of increasing concern. This study investigates the effect of preoperative stroke on short- and long-term outcomes, hypothesizing that it decreases both 1-year mortality and days alive and at home up to 30 days after surgery (DAH30).

**Methods:**

This cohort study investigated 290,306 adult patients with (7214) and without (283,092) preoperative stroke undergoing major non-cardiovascular, non-ambulatory surgery at 23 hospitals in Sweden between 2007 and 2014. Data were pre- and postoperatively matched with quality registers. Using logistic regression, significant independent risk factors influencing the risk of 1-year mortality and impeded DAH30 were identified with adjusted odds ratios calculated.

**Results:**

Preoperative stroke was associated with higher 1-year mortality and lower DAH30, even after full adjustment for other co-morbid and surgical factors.

**Conclusions:**

This large cohort showed preoperative stroke to impact both the patient-centered short-term outcome DAH30 and 1-year mortality. These findings should be considered in perioperative planning.

**Supplementary Information:**

The online version contains supplementary material available at 10.1186/s13741-024-00453-0.

## Introduction

The Lancet Commission on Global Surgery reported an annual total of 313 million surgical procedures in 2015 (Meara et al. 2015). According to an analysis published by the National Institute for Health and Care Research in 2019 at least 4.2 million of the patients undergoing surgery die within 30 days (Nepogodiev et al. 2019). Beyond mortality, insufficient recovery and postoperative complications are common after major surgery (Ou-Young et al. 2023), leading to impeded length of stay (LOS) and Days Alive and at Home up to 30 days after Surgery (DAH30) (Ou-Young et al. 2023; Reilly et al. 2022).

DAH30 incorporates hospital readmissions and is an efficient outcome measure for clinical trials; shown to be significantly lower among high-risk patients and in those suffering postoperative complications (Bell et al. 2019). It is highly sensitive to comorbidity burden (Bell et al. 2019). DAH30 is seen as a patient-centered outcome (Myles et al. 2017) and low DAH30 corresponds to higher hospital costs (Reilly et al. 2022).

One of the more common comorbid conditions is stroke. The lifetime risk of stroke has increased over the last 20 years by 50% and is now one in four people (Collaborators GBDLRoS, Feigin VL, Nguyen G,, et al. 1990). Stroke risk factors include age, comorbidities such as hypertension, diabetes mellitus, high low-density lipoprotein, atrial fibrillation, and hypertriglyceridemia, as well as lifestyle factors such as smoking and physical activity (Shi et al. 2021; Meschia et al. 2014). Stroke primarily affects the elderly population (Statistics on Stroke 2019 [Elektronisk resurs]^2020^,^2019^) and as the population aged over 60 is projected to increase (Luca et al. 2011) this will impact hospital care in general and more specifically perioperative care. There is a relative paucity of data investigating the impact of preoperative stroke on perioperative outcomes (Liao et al. 2014).

Accordingly, the present investigation aimed to describe the impact of preoperative stroke on short- and long-term outcomes. We hypothesized that preoperative stroke would have an impact both on DAH30 and 1-year mortality.

## Patients and methods

The study protocol (2014/1306–31/3) was approved by the Regional Ethics Committee of Stockholm, Sweden. We adhered to the Strengthening of the reporting of observational studies in epidemiology (STROBE) and a checklist can be found in the “Appendices” section.

This observational, registry-based cohort study prospectively collected data from 23 Swedish hospitals. The study population was identified from university-, county- and district hospitals of all levels in Sweden between 2007 and 2014 using the Orbit surgical planning system software, which at the time of the data collection covered approximately 40% of the Swedish population. The Orbit system includes Swedish identity number, patient demographics, American Society of Anaesthesiologists (ASA) physical status classification, date, and duration of anesthesia and surgery.

**Fig. 1:**
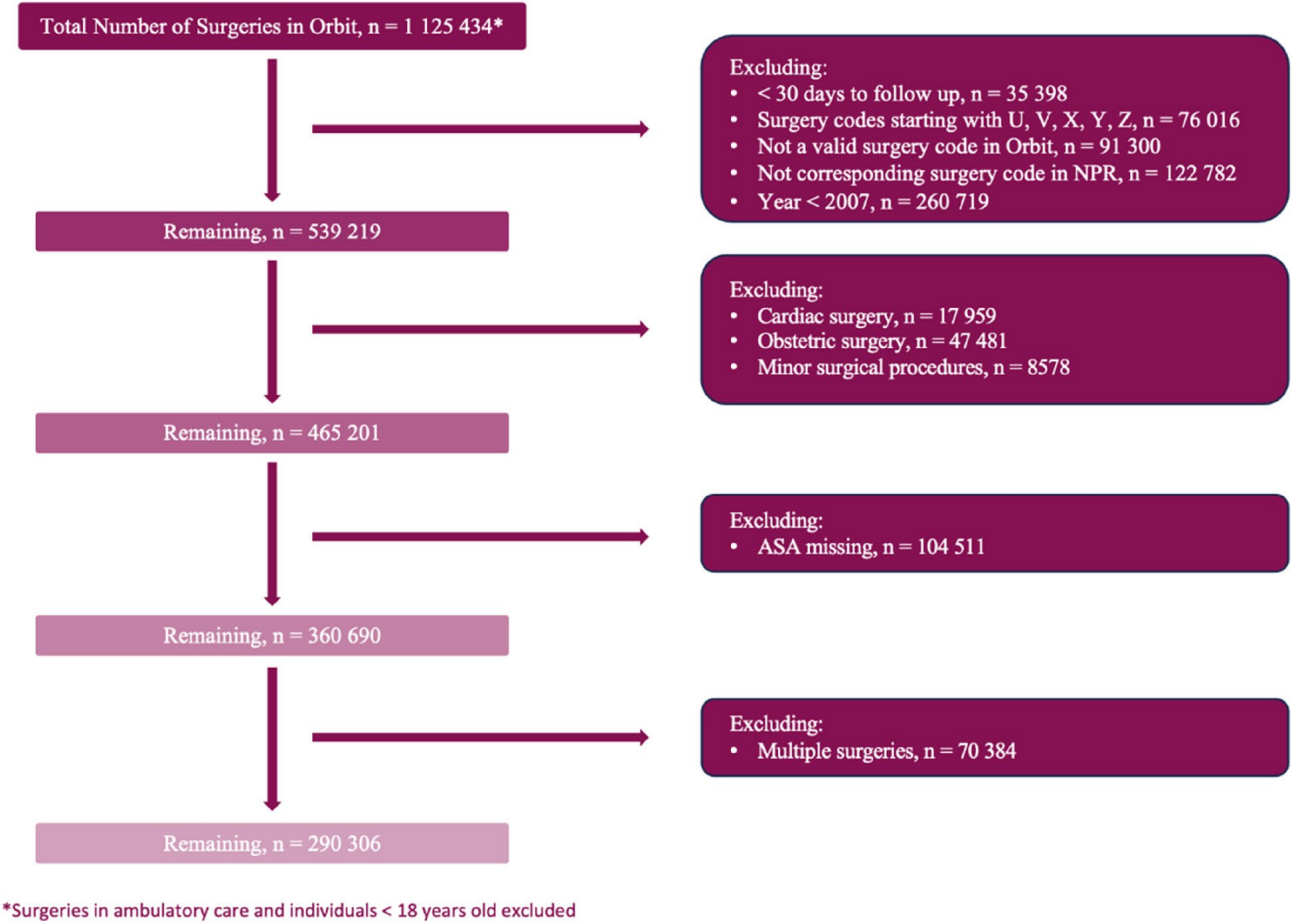
Flowchart

The study cohort included patients ≥ 18 years old who underwent surgery between the 1st of January 2007, and the 31st of December 2014. Exclusions: cardiac, obstetric, ambulatory, minor, or multiple surgeries and those lacking a valid surgery code in Orbit or a corresponding surgery code in the National Patient Register (NPR) (see data sources below). Furthermore, we excluded patients identified from hospitals with a high proportion of missing ASA physical status classification. Figure 1 provides a CONSORT flowchart.

Stroke was defined as all participants with International Statistical Classification of Diseases Code (ICD-code) I63 up to 5 years prior to surgery. In a sensitivity analysis, we defined stroke as ICD-code I63 up to 1 year prior to surgery.

To acquire information on discharge dates, covariates, and drug exposure, surgical records were linked to the National Patient Register (NPR) and the Swedish Prescribed Drug Register (SPDR) using the personal identification number assigned to all residents at birth or immigration. Furthermore, the data was linked to the Swedish Cause of Death Register.

### Data sources

#### Orbit

Orbit is an operational planning system, where all patients undergoing surgery are documented (Care 2023). The Orbit database includes pre-, intra-, and postoperative data.

#### The national patient register

NPR provides statistics on diseases and treatments in Swedish specialized healthcare, covering all in-patient care since 1987 (Socialstyrelsen. 2018a). The NPR comprises personal, geographic, administrative, and medical data, including ICD codes. The registry is obligatory for all Swedish county councils (Socialstyrelsen. 2018a).

#### The Swedish prescribed drug register

The Swedish Prescribed Drug Register (SPDR) provides the basis for the official statistics on pharmaceuticals in Sweden (Socialstyrelsen. 2018b). The register, linked with social security numbers, was initiated in July 2005 and includes all prescription drugs dispensed at pharmacies (Socialstyrelsen. 2018b). The SPDR also consists of information about the patient (such as sex and age), the drug (such as ATC code, dosage, and price) as well as information about the drug prescriber and the workplace where the prescription was made (Socialstyrelsen. 2018b).

#### Swedish cause of death register

The Swedish Cause of Death register is a complete register of all deaths in Sweden since 1952 (Brooke et al. 2017).

### Outcome measures

#### Primary outcome

Our primary outcome was 1-year-mortality in patients with preoperative stroke up to 5 years prior to the operation.

#### Secondary outcome

Our secondary outcome was DAH30 in patients with preoperative stroke up to 5 years prior to the operation. This comprehensive outcome includes length of stay (LOS) and readmissions, as well as mortality within 30 days after surgery.

#### Calculating DAH30

DAH30 was calculated as previously described in detail (Bell et al. 2019; Myles et al. 2017), using the date of index surgery (day 0) with hospitalization and mortality data up to day 30. Hospital—to home—discharge dates were used to calculate hospital length of stay (ignoring preoperative in-hospital days, and taking into account discharge to nursing homes or other hospitals). Death in hospital or after discharge within the first 30 days after surgery, equals 0 DAH30. If a patient was discharged from the hospital on day 13 after surgery but readmitted for 7 days before their second hospital discharge, then the patient would be assigned 10 DAH30.

### Statistical methods

Data were analyzed using RStudio (Team RC. R n.d.). Categorical data were presented as numbers and percentages, while continuous data were presented as means and standard deviations. *P* values < 0.05 were interpreted as statistically significant. Descriptive statistics were presented by *p* values using Pearson’s chi-squared test for binary categorical variables and an unpaired two-sample *t*-test for continuous variables.

To evaluate the primary and secondary outcomes, multiple logistic regression analyses were used. Separate analyses were performed for ASA class and ICD-codes, each one as a proxy for comorbidity, to minimize the risk for collinearity. We adjusted for age, sex, and type of surgery. As mentioned above, a sensitivity analysis was performed among patients with more recent diagnosis of stroke.

DAH_30_ was dichotomized into 1 = DAH ≤ 15, and 0 = DAH > 15. A DAH_30_ less than 15, indicates death and/or complications. This cut-off allows us to calculate adjusted odds ratios (ORs) for preoperative stroke; both for mortality at 365 days and for having a DAH30 less than 15.

## Results

After exclusions, 290,306 patients remained in the study (Fig. 1).

The general characteristics of the population are presented in Table 1. The mean age of the population was 60.1 years (SD 18.9), 45% were men and 44% were classified as ASA 2. Most surgeries (69%) were elective, and orthopedic surgery (32%) was the most common type of surgery.

**Table 1:** Cohort demographics

		**Preoperative stroke**		
**Characteristics**	All (*n* = 290 306)	No (*n* = 283 092)	Yes (*n* = 7214)	*P* value^§^
**Age, years, mean ± SD**	60.1** ± **18.9	59.7 ± 18.9	75.4 ± 11.4	** < 0.001**^**†**^
***Men*, no. (%)**	129 886 (45)	125 998 (45)	3888 (54)	< 0.001^*^
**ASA** Classification**, no. (%)**				
ASA1	85 810 (30)	85 736 (30)	74 (1.0)	< 0.001^*^
ASA2	126 895 (44)	124 617 (44)	2278 (32)	< 0.001^*^
ASA3	71 431 (25)	67 034 (24)	4397 (61)	< 0.001^*^
ASA4	6170 (2.1)	5705 (2.0)	465 (6.4)	< 0.001^*^
**Preoperative data**^**a**^**, no. (%)**				
Heart disease	14 330 (4.9)	13 232 (4.7)	1098 (15)	< 0.001^*^
Renal disease	3057 (1.1)	2878 (1.0)	179 (2.5)	< 0.001^*^
Diabetes mellitus	5342 (1.8)	4913 (1.7)	429 (5.9)	< 0.001^*^
Peripheral vascular disease	3962 (1.4)	3651 (1.3)	311 (4.3)	< 0.001^*^
Cerebrovascular disease	1855 (0.6)	913 (0.3)	942 (13)	< 0.001^*^
Cognitive disease	846 (0.3)	763 (0.3)	83 (1.2)	< 0.001^*^
Substance abuse disease	1096 (0.4)	1052 (0.4)	44 (0.6)	< 0.001^*^
Personality disorder, schizophrenia	1311 (0.5)	1286 (0.5)	25 (0.3)	0.18^*^
Affective disorders	1334 (0.5)	1293 (0.5)	41 (0.6)	0.17^*^
Anxiety disorders	1138 (0.4)	1104 (0.4)	34 (0.5)	0.28^*^
Lung disease	2172 (0.7)	2028 (0.7)	144 (2.0)	< 0.001^*^
Infection	331 (0.1)	318 (0.1)	13 (0.2)	0.092^*^
**Year of surgery, year (%)**				
2007–2010	120 308 (41)	117 228 (41)	3080 (43)	0.029^*^
2011–2014	169 998 (59)	165 864 (59)	4134 (57)	0.029^*^
**Type of surgery, no. (%)**				
Acute	90 247 (31)	87 214 (31)	3 033 (42)	< 0.001^*^
Cancer	61 024 (21)	59 551 (21)	1473 (20)	0.20^*^
Neuro	22 001 (7.6)	21 318 (7.5)	683 (9.5)	< 0.001^*^
Endocrine	8609 (3.0)	8533 (3.0)	76 (1.1)	< 0.001^*^
Ophthalmic	3762 (1.3)	3677 (1.3)	85 (1.2)	0.37^*^
Ear, nose, and throat	7792 (2.7)	7698 (2.7)	94 (1.3)	< 0.001^*^
Oral and maxillofacial	10 403 (3.6)	10 312 (3.6)	91 (1.3)	< 0.001^*^
Lung	3223 (1.1)	3150 (1.1)	73 (1.0)	0.42^*^
Breast	14 381 (5.0)	14 232 (5.0)	149 (2.1)	< 0.001^*^
Abdominal	56 841 (20)	55 785 (20)	1056 (15)	< 0.001^*^
Urologic	29 452 (10)	28 654 (10)	798 (11)	0.009^*^
Gynecologic	22 117 (7.6)	21 886 (7.7)	231 (3.2)	< 0.001^*^
Orthopedic	91 874 (32)	89 044 (31)	2830 (39)	< 0.001^*^
Vascular	11 845 (4.1)	11 007 (3.9)	838 (12)	< 0.001^*^
Skin	8006 (2.8)	7796 (2.8)	210 (2.9)	0.42^*^
**Mortality, no. (%)**				
30-day mortality	4769 (1.6)	4426 (1.6)	343 (4.8)	< 0.001^*^
90-day mortality	9015 (3.1)	8334 (2.9)	681 (9.4)	< 0.001^*^
365-day morality	18 960 (6.5)	17 667 (6.2)	1293 (18)	< 0.001^*^
**Days at home, mean ± SD**				
DAH_30_	24.3 ± 7.4	24.3 ± 7.3	20.2 ± 9.4	** < 0.001**^**†**^
DAH_90_	81.7 ± 15.3	81.8 ± 15.1	74.8 ± 20.8	** < 0.001**^**†**^

*Abbreviations*: *ASA* American Society of Anesthesiologists, *SD* Standard deviation, *DAH30* Days at Home up to 30 days after Surgery, *DAH90* Days at Home up to 90 days after Surgery

^*^Pearson’s chi-squared test

^†^Unpaired two-sample *t* test

^§^Significant *p* values are bolded

^a^ICD-code valid 0–30 days prior to surgery

Of the patients with preoperative stroke up to 5 years before surgery, the mean age was 74.5 (SD 11.4) and 54% were men. Compared to the non-preoperative stroke group, the preoperative stroke group exhibited a higher proportion of individuals classified as ASA 3 and 4. Diabetes mellitus, peripheral vascular disease, and heart disease were more common among individuals with previous strokes. Mortality was higher, and mean DAH_30_ was lower among patients with preoperative stroke.

Table 2 presents the results of the multivariable logistic regression analysis using ASA to adjust for comorbidity with 365-day mortality as an endpoint.

**Table 2:** Multivariable logistic regression with predictors of 365-day mortality using ASA as a proxy for comorbidity

Characteristics	Crude OR, mortality ± 95% CI	Adjusted OR, mortality ± 95% CI	*P* value, OR, mortality^§^ (Wald’s test)
**Preoperative stroke**	3.28 (3.08, 3.49)	1.16 (1.08, 1.24)	** < 0.001**
***Age***			
≤ 39	1.00 (reference)	1.00 (reference)	(reference)
40–49	2.24 (1.92, 2.61)	2.01 (1.72, 2.35)	** < 0.001**
50–59	5.05 (4.44, 5.77)	3.77 (3.30, 4.33)	** < 0.001**
60–69	8.74 (7.74, 9.90)	5.66 (4.99, 6.44)	** < 0.001**
70–79	15.04 (13.34, 17.02)	8.01 (7.07, 9.11)	** < 0.001**
80–89	36.56 (32.46, 41.35)	15.15 (13.37, 17.23)	** < 0.001**
≥ 90	97.26 (85.89, 110.57)	33.62 (29.46, 38.50)	** < 0.001**
**Male sex**	1.26 (1.23, 1.30)	1.19 (1.15, 1.24)	** < 0.001**
***ASA Classification***			
ASA 1	1.00 (reference)	1.00 (reference)	(reference)
ASA 2	6.24 (5.71, 6.84)	3.11 (2.83, 3.41)	** < 0.001**
ASA 3	29.32 (26.89, 32.03)	9.30 (8.48, 10.21)	** < 0.001**
ASA 4	114.23 (103.55, 126.23)	29.07 (26.18, 32.32)	** < 0.001**
**Acute surgery**	3.20 (3.11, 3.30)	2.09 (2.02, 2.17)	** < 0.001**
***Type of surgery***			
Vascular	1.00 (reference)	1.00 (reference)	(reference)
Breast	0.15 (0.13, 0.18)	0.63 (0.54, 0.73)	** < 0.001**
Abdominal	0.88 (0.82, 0.94)	1.67 (1.55, 1.80)	** < 0.001**
Endocrine	0.12 (0.10, 0.15)	0.47 (0.38, 0.58)	** < 0.001**
Gynecologic	0.20 (0.18, 0.23)	0.84 (0.75, 0.95)	**0.006**
Dermatologic	0.74 (0.66, 0.82)	1.18 (1.05, 1.33)	**0.004**
Oral and maxillofacial	0.23 (0.19, 0.26)	1.18 (1.01, 1.37)	**0.034**
Lung	1.66 (1.48, 1.86)	2.18 (1.91, 2.47)	** < 0.001**
Neuro	0.99 (0.92, 1.07)	1.43 (1.31, 1.55)	** < 0.001**
Ophthalmic	0.25 (0.20, 0.31)	0.52 (0.42, 0.65)	** < 0.001**
Ear, nose, and throat	0.15 (0.12, 0.18)	0.60 (0.49, 0.72)	** < 0.001**
Orthopedic	0.83 (0.77, 0.88)	0.83 (0.77, 0.89)	** < 0.001**
Urologic	0.61 (0.57, 0.66)	1.14 (1.05, 1.24)	**0.002**

*Abbreviations*: *ASA* American Society of Anesthesiologists, *OR* Odds ratio, *CI* Confidence interval

^§^Significant *P* values are bolded

The unadjusted ORs for 365 mortality was 3.28 (95% CI 3.08–3.49) but 1.16 (95% CI 1.08–1.24) after full adjustment. Increasing age, male sex, higher ASA class, and acute surgery were associated with the risk of death.

Table 3 shows the secondary outcome, DAH30, dichotomized. The fully adjusted risk for patients with preoperative stroke to have impeded DAH30, i.e., DAH ≤ 15 was 1.09 (95% CI 1.03–1.16). Again, age, male sex, higher ASA class, and acute surgery were associated with this outcome.

**Table 3:** Multivariable logistic regression with predictors of DAH ≤ 15 using ASA as a proxy for comorbidity

**Characteristics**	**Crude** **OR, DAH** ≤ **15** ** ± 95% CI**	**Adjusted OR, DAH** ≤ **15** ** ± 95% CI**	***P* value, OR, DAH** ≤ **15**^**§**^ **(Wald’s test)**
**Preoperative stroke**	2.67 (2.53, 2.82)	1.09 (1.03, 1.16)	**0.005**
***Age***			
≤ 39	1.00 (reference)	1.00 (reference)	(reference)
40–49	1.43 (1.34, 1.52)	1.28 (1.20, 1.38)	** < 0.001**
50–59	1.95 (1.84, 2.06)	1.47 (1.38, 1.57)	** < 0.001**
60–69	2.48 (2.35, 2.61)	1.66 (1.57, 1.76)	** < 0.001**
70–79	3.67 (3.49, 3.86)	2.02 (1.90, 2.14)	** < 0.001**
80–89	7.00 (6.66, 7.37)	2.85 (2.68, 3.02)	** < 0.001**
≥ 90	10.69 (10.02, 11.40)	2.97 (2.76, 3.20)	** < 0.001**
**Male sex**	1.24 (1.21, 1.27)	1.08 (1.06, 1.11)	** < 0.001**
***ASA Classification***			
ASA 1	1.00 (reference)	1.00 (reference)	(reference)
ASA 2	3.64 (3.47, 3.83)	2.83 (2.69, 2.99)	** < 0.001**
ASA 3	14.81 (14.10, 15.55)	7.93 (7.51, 8.37)	** < 0.001**
ASA 4	65.24 (60.93, 69.88)	26.91 (24.98, 29.00)	** < 0.001**
**Acute surgery**	3.20 (3.13, 3.28)	2.22 (2.16, 2.28)	** < 0.001**
***Type of surgery***			
Vascular	1.00 (reference)	1.00 (reference)	(reference)
Breast	0.03 (0.03, 0.04)	0.10 (0.08, 0.13)	** < 0.001**
Abdominal	0.98 (0.92, 1.04)	1.57 (1.47, 1.67)	** < 0.001**
Endocrine	0.08 (0.07, 0.10)	0.22 (0.18, 0.27)	** < 0.001**
Gynecologic	0.17 (0.15, 0.18)	0.50 (0.45, 0.56)	** < 0.001**
Dermatologic	1.05 (0.96, 1.14)	1.69 (1.54, 1.84)	** < 0.001**
Oral and maxillofacial	0.17 (0.15, 0.19)	0.58 (0.50, 0.67)	** < 0.001**
Lung	2.37 (2.15, 2.60)	2.71 (2.44, 3.01)	** < 0.001**
Neuro	2.66 (2.50, 2.82)	3.70 (3.46, 3.95)	** < 0.001**
Ophthalmic	0.10 (0.07, 0.12)	0.17 (0.13, 0.22)	** < 0.001**
Ear, nose, and throat	0.16 (0.14, 0.19)	0.48 (0.41, 0.56)	** < 0.001**
Orthopedic	1.04 (0.98, 1.10)	1.22 (1.15, 1.30)	** < 0.001**
Urologic	0.34 (0.32, 0.37)	0.59 (0.54, 0.64)	** < 0.001**

*Abbreviations*: *ASA* American Society of Anesthesiologists, *OR* Odds Ratio, *CI* Confidence interval

^§^Significant *P* values are bolded

In the “Appendices” section, Supplementary Tables 1 and 2 show the primary and secondary outcomes, 365-day mortality, and DAH30 when instead using ICD-10 codes to adjust for comorbidity. In both analyses, the association of having a stroke during 5 years prior to surgery with 1-year mortality or impeded DAH30 appears to be highly significant.

## Discussion

In this national cohort study of close to 300,000 patients, those with preoperative stroke undergoing major surgery had an elevated risk of mortality in the postoperative 1-year follow-up. Importantly, preoperative stroke was also associated with a lower number of days at home and alive.

The present study adds novel data with regards to the endpoints chosen: it is uncommon to report 1-year mortality, and even though similar metrics have been used for stroke, this is the first time the novel patient-centered composite endpoint, DAH30, has been used in this setting (Bell et al. 2019). However, there are similarities to an investigation using Taiwan’s National Health Insurance Research Database; patients with previous stroke who underwent surgery had an increased risk of postoperative pneumonia, septicemia, acute renal failure, acute myocardial infarction, pulmonary embolism, and 30-day in-hospital mortality (Liao et al. 2014). Those complications are known to lead to increased length of stay and/or risk for readmissions—lowering DAH30. Notably, the Taiwanese study defined previous stroke as taking place within 2 years before index surgery, whereas we defined it as taking place within 5 years before the operation. Time from stroke to surgery is important: risk of major adverse cardiovascular events (MACE; ischemic stroke, acute myocardial infarction, and cardiovascular mortality) and all-cause mortality up to 30 days after surgery has previously been shown to be associated with time elapsed after ischemic stroke and elective surgery (Jorgensen et al. 2014a). Compared with patients without stroke, a Danish nationwide study reported ORs for MACE at 14.23 for stroke less than 3 months prior to surgery, 4.85 for stroke 3 to less than 6 months prior, 3.04 for stroke 6 to less than 12 months prior, and 2.47 for stroke 12 months or more prior. All-cause mortality followed a similar pattern, with the highest risk in the group with the shortest time elapsed between the ischemic stroke and the surgical event. The study from Denmark raised questions. Does surgery increase the risk of recurrent stroke? Or would recurrent stroke have occurred at comparable rates among patients not undergoing surgery? This was addressed by the authors in follow-up analyses, confirming a pattern of temporal decline in the risk of recurrent stroke for the background stroke population but with absolute rates much less pronounced than for the surgery group (Jorgensen et al. 2014b).

Our findings have medical consequences. The fact that patients with preoperative stroke have lowered DAH30 as well as increased long-term mortality should lead to a reckoning with regard to surgical and anesthetic planning. In fact, the present results regarding short- and long-term trajectories following surgery in patients with previous strokes add to the opportunities for shared decision-making (Shaw et al. 2023). This is especially important in older high-risk patients offered major surgery. Notably, in both patients with stroke up to 5 years prior to the surgery, and in patients with a diagnosis of stroke 1 year before the operation, risks of adverse outcomes are elevated. The fact that adjusting for ASA class (as compared to comorbid adjustment using ICD-codes, as seen in the supplement) performs “better” likely has to do with the fact that the ASA classification is done quite close to the planned surgery, leading to more up to date comorbidity information.

This investigation has strengths and weaknesses. We lack data on the type of anesthesia, previous propensity score matched data indicate that stroke patients undergoing general anesthesia have increased postoperative complications and mortality after surgery compared with those who received neuraxial anesthesia (Kao et al. 2022). We have no physiological intraoperative information, precluding analysis of potentially modifiable factors, such as avoidance of hypotension. Information on patient frailty is missing and could likely improve our ability to detect if preoperative stroke is an independent risk factor. Excluding patients with missing ASA class is a weakness, however, this subset of patients has been demonstrated in previous studies to have almost identical properties in terms of demographics as well as identical outcomes in terms of mortality (Hallqvist et al. 2020). This perioperative database has data up to 2015, so general improvements since then are not accounted for. Strengths include that we report high-resolution and complete perioperative data across smaller regional institutions to major university hospitals. Using day-by-day postoperative hospitalization data allowed us to investigate the postsurgical outcome metric DAH30, used for the first time to characterize the impact of preoperative stroke. Cohort size permits us to adjust for multiple covariates. Finally, overall generalizability on the impact of preoperative stroke should be decent in industrialized nations.

## Conclusion

In conclusion, we find that patients with stroke during 5 years prior to surgery are at increased risk of adverse short- and long-term outcomes. This is indicated by lowered DAH30 and higher 1-year mortality. These findings should be considered when planning surgery for this patient population.

## Supplementary Information

Supplementary Material 1.

## Data Availability

No datasets were generated or analysed during the current study.
