# Correction: The impact of preoperative stroke on 1-year mortality and days at home alive after major surgery: an observational cohort study

**DOI:** 10.1186/s13741-024-00467-8

**Published:** 2024-11-12

**Authors:** Matilda Widaeus, Alva Cedermark, Max Bell

**Affiliations:** 1 Department of Anaesthesia and Intensive Care Medicine, Karolinska University Hospital, Stockholm, Sweden; 2 Department of Physiology and Pharmacology, Karolinska Institute, Stockholm, Sweden

**Correction: Perioper Med 13, 97 (2024)**

**https://doi.org/10.1186/s13741-024-00453-0**

Following publication of the original article (Widaeus et al. 2024), the authors identified an error in the author name of Alva Cedermark.

The incorrect author name is: Alva Cederlund

The correct author name is: Alva Cedermark

The author group has been updated above and the original article (Widaeus et al. 2024) has been corrected.
