# Supplementary material for: The impact of preoperative stroke on 1-year mortality and days at home alive after major surgery: an observational cohort study
Source: Perioper Med (Lond). 2024 Oct 4;13:97. doi: 10.1186/s13741-024-00453-0 (PMC11451116; doi:10.1186/s13741-024-00453-0)
Supplement: Supplementary file 1 — Supplementary Material 1. [file 13741_2024_453_MOESM1_ESM.docx]

**APPENDIX**

**Supplementary table 1. Multivariable logistic regression with predictors of 365-day mortality using ICD-10 codes as proxy for comorbidity.**

| Characteristics | Crude  OR, mortality  ± 95% CI | Adjusted OR, mortality  ± 95% CI | P-value, OR,  mortality^§^  (Wald’s test) |
| --- | --- | --- | --- |
| Preoperative stroke | 3.28 (3.08, 3.49) | 1.46 (1.36, 1.57) | **< 0.001** |
| *Age* |  |  |  |
| ≤ 39 | 1.00 (reference) | 1.00 (reference) | (reference) |
| 40-49 | 2.24 (1.92, 2.61) | 2.70 (2.32, 3.15) | **< 0.001** |
| 50-59 | 5.05 (4.44, 5.77) | 6.45 (5.66, 7.37) | **< 0.001** |
| 60-69 | 8.74 (7.74, 9.90) | 11.46 (10.13, 13.01) | **< 0.001** |
| 70-79 | 15.04 (13.34, 17.02) | 18.98 (16.81, 21.52) | **< 0.001** |
| 80-89 | 36.56 (32.46, 41.35) | 42.18 (37.37, 47.81) | **< 0.001** |
| ≥ 90 | 97.26 (85.89, 110.57) | 106.79 (93.99, 121.80) | **< 0.001** |
| Male sex | 1.26 (1.23, 1.30) | 1.25 (1.21, 1.29) | **< 0.001** |
| *Preoperative data*^‡^ |  |  |  |
| Heart disease | 3.21 (3.06, 3.36) | 1.44 (1.36, 1.53) | **< 0.001** |
| Renal disease | 3.78 (3.46, 4.13) | 2.45 (2.20, 2.72) | **< 0.001** |
| Diabetes Mellitus | 3.23 (3.00, 3.47) | 1.59 (1.46, 1.73) | **< 0.001** |
| Peripheral vascular disease | 3.21 (2.95, 3.48) | 1.41 (1.27, 1.55) | **< 0.001** |
| Cerebrovascular disease | 3.20 (2.84, 3.60) | 0.82 (0.71, 0.94) | **0.006** |
| Cognitive disease | 6.64 (5.73, 7.68) | 1.80 (1.53, 2.12) | **< 0.001** |
| Substance abuse disease | 1.58 (1.29, 1.92) | 1.51 (1.21, 1.87) | **< 0.001** |
| Personality disorder, schizophrenia | 0.84 (0.66, 1.06) | 1.93 (1.47, 2.51) | **< 0.001** |
| Affective disorders | 1.25 (1.02, 1.52) | 1.19 (0.95, 1.48) | 0.123 |
| Anxiety disorders | 1.08 (0.86, 1.35) | 1.50 (1.15, 1.94) | **0.002** |
| Lung disease | 6.03 (5.48, 6.62) | 2.37 (2.12, 2.64) | **< 0.001** |
| Infection | 4.21 (3.23, 5.41) | 1.82 (1.35, 2.43) | **< 0.001** |
| Acute surgery | 3.20 (3.11, 3.30) | 2.59 (2.50, 2.69) | **< 0.001** |
| *Type of surgery* |  |  |  |
| Vascular | 1.00 (reference) | 1.00 (reference) | (reference) |
| Breast | 0.15 (0.13, 0.18) | 0.48 (0.41, 0.56) | **< 0.001** |
| Abdominal | 0.88 (0.82, 0.94) | 1.49 (1.37, 1.61) | **< 0.001** |
| Endocrine | 0.12 (0.10, 0.15) | 0.45 (0.36, 0.55) | **< 0.001** |
| Gynecologic | 0.20 (0.18, 0.23) | 0.67 (0.60, 0.76) | **< 0.001** |
| Dermatologic | 0.74 (0.66, 0.82) | 1.14 (1.01, 1.28) | **0.031** |
| Oral and maxillofacial | 0.23 (0.19, 0.26) | 0.97 (0.83, 1.13) | 0.722 |
| Lung | 1.66 (1.48, 1.86) | 3.05 (2.67, 3.47) | **< 0.001** |
| Neuro | 0.99 (0.92, 1.07) | 1.83 (1.67, 1.99) | **< 0.001** |
| Opthalmic | 0.25 (0.20, 0.31) | 0.39 (0.31, 0.49) | **< 0.001** |
| Ear, Nose and Throat | 0.15 (0.12, 0.18) | 0.50 (0.41, 0.61) | **< 0.001** |
| Orthopedic | 0.83 (0.77, 0.88) | 0.69 (0.64, 0.75) | **< 0.001** |
| Urologic | 0.61 (0.57, 0.66) | 0.89 (0.82, 0.98) | **0.012** |
| Abbreviations: ASA = American Society of Anesthesiologists, OR = Odds Ratio, CI = Confidence Interval. ^‡^ ICD-code valid 0-30 days prior to surgery. ^§^ Significant P-values bolded. | | | |

**Supplementary table 2. Multivariable logistic regression with predictors of DAH≤15 using ICD-10 codes as proxy for comorbidity.**

| Characteristics | Crude  OR, DAH≤15  ± 95% CI | Adjusted OR, DAH≤15  ± 95% CI | P-value, OR,  DAH≤15^§^  (Wald’s test) |
| --- | --- | --- | --- |
| Preoperative stroke | 2.67 (2.53, 2.82) | 1.40 (1.32, 1.49) | **< 0.001** |
| *Age* |  |  |  |
| ≤ 39 | 1.00 (reference) | 1.00 (reference) | (reference) |
| 40-49 | 1.43 (1.34, 1.52) | 1.65 (1.54, 1.76) | **< 0.001** |
| 50-59 | 1.95 (1.84, 2.06) | 2.37 (2.24, 2.52) | **< 0.001** |
| 60-69 | 2.48 (2.35, 2.61) | 3.19 (3.02, 3.37) | **< 0.001** |
| 70-79 | 3.67 (3.49, 3.86) | 4.53 (4.29, 4.78) | **< 0.001** |
| 80-89 | 7.00 (6.66, 7.37) | 7.66 (7.26, 8.10) | **< 0.001** |
| ≥ 90 | 10.69 (10.02, 11.40) | 9.58 (8.94, 10.26) | **< 0.001** |
| Male sex | 1.24 (1.21, 1.27) | 1.12 (1.09, 1.15) | **< 0.001** |
| *Preoperative data*^‡^ |  |  |  |
| Heart disease | 2.42 (2.32, 2.52) | 1.26 (1.19, 1.32) | **< 0.001** |
| Renal disease | 2.48 (2.28, 2.70) | 2.01 (1.81, 2.21) | **< 0.001** |
| Diabetes Mellitus | 2.90 (2.73, 3.09) | 1.62 (1.50, 1.74) | **< 0.001** |
| Peripheral vascular disease | 3.60 (3.36, 3.85) | 2.25 (2.07, 2.44) | **< 0.001** |
| Cerebrovascular disease | 3.42 (3.10, 3.78) | 0.94 (0.84, 1.06) | 0.340 |
| Cognitive disease | 3.65 (3.15, 4.22) | 1.10 (0.94, 1.30) | 0.226 |
| Substance abuse disease | 2.80 (2.44, 3.20) | 1.98 (1.69, 2.30) | **< 0.001** |
| Personality disorder, schizophrenia | 1.78 (1.54, 2.04) | 2.95 (2.50, 3.48) | **< 0.001** |
| Affective disorders | 1.74 (1.51, 2.00) | 1.48 (1.26, 1.74) | **< 0.001** |
| Anxiety disorders | 1.60 (1.37, 1.87) | 1.61 (1.34, 1.93) | **< 0.001** |
| Lung disease | 4.30 (3.94, 4.70) | 1.90 (1.71, 2.10) | **< 0.001** |
| Infection | 3.95 (3.13, 4.96) | 2.55 (1.96, 3.31) | **< 0.001** |
| Acute surgery | 3.20 (3.13, 3.28) | 2.60 (2.53, 2.67) | **< 0.001** |
| *Type of surgery* |  |  |  |
| Vascular | 1.00 (reference) | 1.00 (reference) | (reference) |
| Breast | 0.03 (0.14, 0.16) | 0.08 (0.07, 0.11) | **< 0.001** |
| Abdominal | 0.98 (0.92, 1.04) | 1.52 (1.42, 1.62) | **< 0.001** |
| Endocrine | 0.08 (0.07, 0.10) | 0.23 (0.19, 0.28) | **< 0.001** |
| Gynecologic | 0.17 (0.15, 0.18) | 0.43 (0.39, 0.48) | **< 0.001** |
| Dermatologic | 1.05 (0.96, 1.14) | 1.69 (1.55, 1.85) | **< 0.001** |
| Oral and maxillofacial | 0.17 (0.14, 0.19) | 0.52 (0.45, 0.59) | **< 0.001** |
| Lung | 2.37 (2.15, 2.60) | 4.12 (3.71, 4.57) | **< 0.001** |
| Neuro | 2.66 (2.50, 2.82) | 5.07 (4.73, 5.44) | **< 0.001** |
| Opthalmic | 0.10 (0.07, 0.12) | 0.14 (0.11, 0.19) | **< 0.001** |
| Ear, Nose and Throat | 0.16 (0.14, 0.19) | 0.44 (0.37, 0.51) | **< 0.001** |
| Orthopedic | 1.04 (0.98, 1.10) | 1.09 (1.02, 1.17) | **0.009** |
| Urologic | 0.34 (0.32, 0.37) | 0.52 (0.48, 0.57) | **< 0.001** |
| Abbreviations: ASA = American Society of Anesthesiologists, OR = Odds Ratio, CI = Confidence Interval. ^‡^ ICD-code valid 0-30 days prior to surgery. ^§^ Significant P-values bolded. | | | |
